# Synergistic and Antagonistic Effects of Biogenic Silver Nanoparticles in Combination With Antibiotics Against Some Pathogenic Microbes

**DOI:** 10.3389/fbioe.2021.652362

**Published:** 2021-04-20

**Authors:** Kawther Aabed, Afrah E. Mohammed

**Affiliations:** Department of Biology, College of Science, Princess Nourah Bint Abdulrahman University, Riyadh, Saudi Arabia

**Keywords:** *Artemisia absinthium*, antimicrobial, cytotoxicity, *Anastatica hierochuntica*, silver nanoparticle

## Abstract

The latest advances in green nanoparticle synthesis have preserved natural and non-renewable resources and decreased environmental pollution. The current study was designed to evaluate silver nanoparticles (AgNPs) fabricated using aqueous extracts of two medicinal plants, *Anastatica hierochuntica* L. (Kaff Maryam) and *Artemisia absinthium*. The phytochemicals were detected by Fourier-transform infrared spectroscopy (FTIR) and Chromatography/Mass Spectrometry (GC-MS). The effects of the AgNPs on *Pseudomonas aeruginosa, Escherichia coli*, *Staphylococcus aureus*, and *Candida albicans* as well as the cytotoxicity against MDA-MB-231 cells were examined. The synergistic and antagonistic effects of the biogenic AgNPs in combination with standard antibiotics against several microbes were also investigated. The ability of the plant extracts to transfer silver ions to AgNPs was measured via dynamic light scattering, zeta potential measurement, and transmission electron microscopy. The most sensitive microbes to AgNP treatment were examined via scanning electron microscopy to assess morphological changes. Biogenic AgNPs showed significant antibacterial effects against most of the tested microbes and significant cytotoxicity was noted. Polysaccharides, proteins and Phenolic compounds are likely involved in AgNP biosynthesis since hydroxyl groups and amides were detected via FTIR as well as GC-MS. This study confirmed that plant-based AgNP fabrication with AgNO_3_ as the Ag (I) delivering salt can be an economical and practical approach for large-scale production of particles with antimicrobial and cytotoxic potential. The synergistic effects of biogenic AgNPs in combination with some antibiotics support their potential as a safe therapeutic for bacterial infections because they are capped with organic biomolecules.

## Introduction

Recently, nanotechnology has emerged as a rapidly developing research field in materials science with the potential to positively affect human health (Singh et al., 2010). In nanotechnology, silver nanoparticles (AgNPs) are the most important metal element with beneficial properties. AgNPs ranging in diameter from 1 to 100 nm are an attractive research target due to their antimicrobial and cytotoxic potential imparted by their ability to easily attach to the cell wall. This attachment leads to effects on cellular respiration and permeability that result in cell death. Furthermore, AgNPs can also easily enter cells to interact with biomolecules, including DNA and protein via their phosphorus and sulfur groups, respectively. Because silver ions have been shown to have no significant effects on cell viability, silver-containing materials can be used in textiles and as additives in food and packaging to eliminate microbes. Due to their varied useful applications as antimicrobial and cytotoxic agents, multiple forms of silver, including silver ions and silver nanoparticles, have been developed (David et al., 2010). Silver ions have been successfully used as antimicrobial agents, although AgNPs have shown higher antimicrobial activity in comparative studies. Very small quantities of AgNPs may have higher antimicrobial effects compared to that for their bulk material. The increased prevalence of antibiotic-resistant microbes, currently considered a widespread health problem, is likely related to intensifying antibiotic use. Decreased antibiotic efficacy has been clearly documented (Huh and Kwon, 2011; El-Mokhtar and Hetta, 2018; El-Baky et al., 2019), triggering a surge in investigations into the antibacterial properties of other materials. Considerable attempts to develop antibiotic alternatives to prevent the rise of antibiotic-resistant microbes has highlighted the potential of nanomaterials. Silver nanoparticles are a promising approach for fighting microbial resistance since microbes are unlikely to accumulate the numerous mutations required to acquire nanoparticle resistance (Friedman et al., 2013). The delivery system for nano drugs involves the production, synthesis, and application of nanomaterials with sizes ranging from 1 to 100 nm (Madhuri et al., 2012; Abd Ellah et al., 2019a, b). Preparation of nanomaterials using chemical and physical approaches has multiple disadvantages, including the generation of toxic chemical byproducts and high energy consumption, making biological synthesis an excellent alternative. Biological systems for nanoparticle production have key advantages over other systems, including the production of more stable nanoparticles and increased control over size and shape. These advantages support the production of biogenic nanoparticles appropriate for various nanotechnology applications and provide nanoparticles with high compatibility for biomedical purposes since no toxic chemicals are used in their production. Various benign environmental agents have been used for AgNP production, including plant extract (Jain et al., 2009), fungi (Verma et al., 2010), bacteria (Saifuddin et al., 2009), and enzymes (Willner et al., 2007). Plants are a well known source of antimicrobial and antioxidant agents (Lewis and Ausubel, 2006; Tegos, 2006). In the current study, based on their various pharmacological applications, *Anastatica hierochuntica* and *Artemisia absinthium* have been used for AgNP fabrication. *A. hierochuntica* extracts showed antimicrobial, antifungal, antioxidant, hypolipidemic, and hypoglycemic effects (Rahmy and El-Ridi, 2002; Tayel and El-Tras, 2009; Mohamed et al., 2010; Al Gamdi et al., 2011; Salah et al., 2011; Daoowd, 2013; Abou-Elella et al., 2016). *A. absinthium*, which is as the main component in the infamous Absinthe drink, has been known for its medicinal benefits since the time of the ancient Greeks and in traditional medicine of western Europe. Furthermore, antimicrobial properties of essential oils from the flowers and aerial parts of *A. absinthium* are well documented (Juteau et al., 2003). *A. absinthium* contains caffeoyl and dicaffeoylquinic acids, which can prevent HIV-1 integrase from incorporating viral DNA into the host genome (Reinke et al., 2002). These properties inspired our rationale for using these plant sources as biomediators in AgNP formation with the expectation that the resulting AgNPs would have higher antimicrobial activity than the plant extracts and silver ions alone. Relevant to the increasing prevalence of antibiotic resistance, combinations of nanoparticles and antibiotics have been shown to have higher efficacy relative to antibiotics alone in clinical settings (Abo-Shama et al., 2020). Such combinations could limit the development of antibiotic resistance and reduce the duration and dose requirements for antibiotic treatment (Hwang et al., 2012a). Based on this framework, the current report describes the fabrication of AgNPs using two medicinal plants as biogenic agents. The efficacy of the resulting AgNPs in inhibiting the growth of bacterial and fungal pathogens alone and in combination with antibiotics was also investigated.

## Materials and Methods

### Materials

Silver nitrate (AgNO_3_) was obtained from Saudi Overseas Marketing and Trading Company (SOMATCO), Riyadh, Saudi Arabia. Nutrient agar plates and nutrient broth (Difco, Becton, Dickinson and Company, Sparks Glencoe, MD, United States) as well as potato-dextrose agar (PDA) plates (Difco, Becton, Dickinson and Company, Sparks Glencoe, MD, United States) were purchased from Wateen Alhaiah Company, Riyadh, Saudi Arabia. All clinical bacterial isolates were obtained from the Microbiology Laboratory of Princess Nourah bint Abdulrahman University, Riyadh, Saudi Arabia. Antibiotic discs were obtained from OXOID ^TM^ United Kingdom at the following concentrations: bacitracin, 10 μg/mL; ciprofloxacin, 10 μg/mL; tetracycline, 30 μg/mL; and cefixime, 5 μg/mL.

### Plant Materials

Complete *A. hierochuntica* L. (Kaff Maryam) plants and *A. absinthium* seeds were obtained from a local market in Riyadh, Saudi Arabia. Their identities were verified, and they were stored in polythene bags at 4°C prior to use. Plant parts were cleaned with distilled water, air dried, and then ground into a fine powder using a milling machine (IKA-Werke, GMBH and Co., Germany). The powder was stored at room temperature in plastic bags until use.

### Preparation of Aqueous Extracts for AgNP Fabrication

Aqueous extracts of each plant were prepared via addition of water to the powder at a 10:1 (w:v) ratio. The mixtures were immediately heated to 80°C for 10 min for enzyme deactivation. Next, Whatman Grade No. 1 filter paper (diameter of 125 mm and mean pore size of 11 μm was used to filter the mixtures, and the supernatants were retained for use in AgNP synthesis. For AgNP synthesis, 10 ml of plant extract was combined with 90 ml of AgNO_3_ solution (1 mM) in a flask. The reaction was kept at room temperature in the dark for 48 h until a stable dark color developed. The mixture was then stored at 4°C.

### Dynamic Light Scattering (DLS) and Zeta Potential Measurement

To characterize the prepared AgNPs, a Zetasizer nano device (Nano ZSP, Malvern Instruments Ltd., Serial Number: MAL1118778, ver. 7.11, United Kingdom) was used to determine the size distribution via DLS and the electrical charge via zeta potential measurement.

### Transmission Electron Microscopy (TEM)

Transmission electron microscopy (JEM-1011, JEOL, Japan; 80 kV) was used to examine the AgNP morphology and size distribution. The tested materials were placed on carbon-coated (200 mesh) TEM grids via drop-coating.

### Fourier-Transform Infrared Spectroscopy (FTIR)

Fourier-transform infrared spectroscopy spectroscopy is useful for analyzing the functional groups of biomolecules in a sample by measuring infrared absorption and emission spectra. FTIR (Spectrum100, Perkin-Elmer, United States) was performed with scanning data collected over a range of 450 to 3,500 cm^–1^.

### Microbial Analyses

Four different microbes were used to assess the antimicrobial activity of the biogenic AgNPs (*P. aeruginosa, E. coli, S. aureus*, and *C. albicans*). Microbial cultures were prepared in the Department of Biology, College of Science, Riyadh, KSA.

### Antimicrobial Activity

The antibacterial effects of the biogenic AgNPs were assessed via agar well diffusion assays (Perez et al., 1990). Approximately 20 ml of Mueller-Hinton agar were placed in sterilized Petri dishes. Bacteria were cultured in nutrient broth for 24 h at 37°C. Approximately 0.2 ml of nutrient broth (containing 10^8^ CFU/ml) were used to prepare bacterial lawns. On each agar plate, four wells were prepared using a sterilized 4-mm cork borer and then filled with the test materials. Sterile distilled water was used as a negative control, and AgNO_3_ was used as a positive control.

The plates were then incubated for 18 h at 37°C for bacteria and for 48 to 96 h for fungi at 28°C; the zones of inhibition around the wells were then measured using a metric ruler and expressed as the mean value (in mm) for each plate (Cheesbrough, 2000).

### Minimum Inhibitory Concentration (MIC) and Minimum Bactericidal Concentration (MBC) Determination

The MIC values were determined using the microdilution method. Approximately 10 μl of each bacterial culture (10^8^ CFU/ml) was combined individually with 10 mL of nutrient broth (NB). Next, AgNPs were added to each tube of bacterial culture at various concentrations followed by incubation for 24 h at 37°C. Thereafter, the MIC values were determined by examining the turbidity in each tube. The MIC was defined as the minimum AgNPs concentration that prevented bacterial growth (Das et al., 2016). The minimum bactericidal concentration (MBC) was defined as the concentration that killed 99.9% of the bacteria (Basri and Fan, 2005).

### Scanning Electron Microscopy (SEM)

Scanning electron microscopy (SEM) (Hitachi S-4500) was used to identify morphological changes to the external cell wall of AgNP-treated microbes. Thin layers of treated microbes were dropped onto carbon-coated copper grids, and the excess solution was removed with blotting paper. The thin layer on the SEM grid was dried by subjecting the grids to a mercury lamp for 5 min.

### Synergistic Antibacterial Activity of AgNPs

Synergistic antibacterial effects of mixtures of AgNP and antibiotics (bacitracin, ciprofloxacin, tetracycline, and cefixime) were examined via a standard disk diffusion method. To assess synergism, bacteria were grown on nutrient agar plates. AgNPs (1 mg/mL) were mixed with each antibiotic (bacitracin, 10 μg/mL; ciprofloxacin, 10 μg/mL; tetracycline, 30 μg/mL; and cefixime, 5 μg/mL) at a 1:1 (v:v) ratio, and the solutions were sonicated at room temperature for 15 min. The synergistic antibacterial effects of the AgNP-antibiotic mixtures were assessed after 24 h of incubation at 37°C based on the diameters of the inhibition zones around the disks (expressed in mm).

### Synergistic Anticandidal Activity of AgNPs

Synergistic anticandidal effects of mixtures of AgNPs and anticandidal agents (fluconazole and metronidazole) were examined via a standard disk diffusion method. AgNPs (2 mg/mL) and anticandidal agents (fluconazole, 150 μg/mL; metronidazole, 125 μg/mL) were combined at a 1:1 (v:v) ratio, and the solutions were sonicated at room temperature for 15 min. *C. albicans* (in liquid medium) was spread uniformly on potato-dextrose agar (PDA) plates. Disks containing the anticandidal agents were then placed on the plates followed by incubation for 48 h at 28°C. Synergistic anticandidal effects of the AgNP-anticandidal agent mixtures were assessed based on the diameters of the inhibition zones around the disks (expressed in mm).

### MTT Assay

A 250 mg of MTT [3-(4,5-dimethylthiazol-2-yl)-2,5-diphenyl tetrazolium bromide] was used to assess cytotoxicity. MDA-MB-231 cells were treated with biogenic AgNPs, and the optical density (OD) was determined by measuring the absorbance at 595 nm using an ELISA reader (Anthos 2010 Microplate Reader, Biochrom Ltd., United Kingdom). Cell viability was calculated using following formula:

Cellviability(in%)=(ODsample/ODcontrol)×100.

The IC_50_ (the half-maximal inhibitory concentration) is the concentration required to inhibit growth by 50%. IC_50_ values were calculated from regression curves.

### Chromatography/Mass Spectrometry (GC-MS) Techniques

The chemical analysis of *A. hierochuntica* and *A. absinthium* was examined by GC-MS (AGILENT Technologies 220 Ion Trap GC/MS, United States) applying the alcoholic extract of both plant sources separately. Helium has been used as the carrier gas with a column (brand?) of the following specifications: Flow rate 1 mL/min; Pressure 8.2317 psi; Average velocity 36.623 cm/s; Holdup flow 1.3653 min; Post run 0.99996 mL/min; Column max. temperature 450°C, 30 m × 250 μm × 0.25 μm). Initial oven temperature was 70°C with run time is at 52 min to reach finally 250°C. The biomolecules were determined using the National Institute of Standards and Technology (NIST) chemical database.

### Statistical Analyses

Results are stated as the mean ± standard deviation (SD) of three independent replicates (calculated using Excel Microsoft Office 2010). The IC_50_ values were computed using GRAPHPAD PRISM 8.1 to produce the cytotoxicity assay plots.

## Results

The current investigation aimed to examine the usefulness of *A. hierochuntica* and *A. absinthium* extracts as biomediators in AgNP formation, and the antibacterial and anticandidal activities of the resulting AgNPs were examined. In the current investigation, aqueous extracts of *A. hierochuntica* and *A. absinthium* were used for AgNP fabrication. Addition of the plant extracts to a solution of AgNO_3_ yielded mixtures that changed from pale yellow to brown-yellow for An-AgNPs and Ar-AgNPs prepared from *A. hierochuntica* (An) (Supplementary Files A,B) and *A. absinthium* (Ar) extracts (Supplementary Files C,D). AgNP biosynthesis proceeded first via a slow color change in the reaction mixture (after approximately 1 h of incubation) after which the color intensity increased over the remainder of the reaction.

### Characterization of Biogenic AgNPs

**FIGURE 1:**
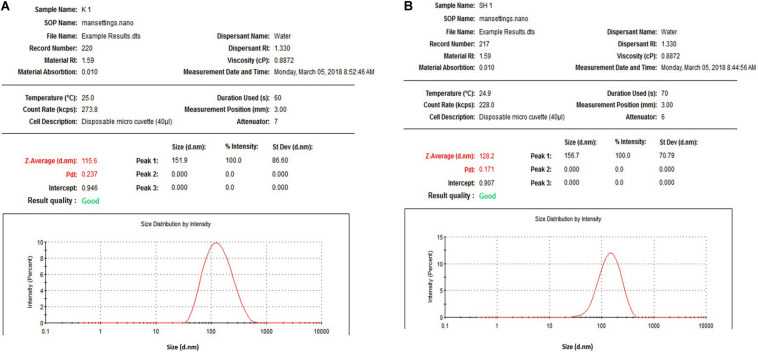
Size distribution of AgNPs obtained using aqueous extracts of *A. hierochuntica* **(A)** and Size distribution of AgNPs obtained using aqueous extracts of *A. absinthium* **(B)**.

**FIGURE 2:**
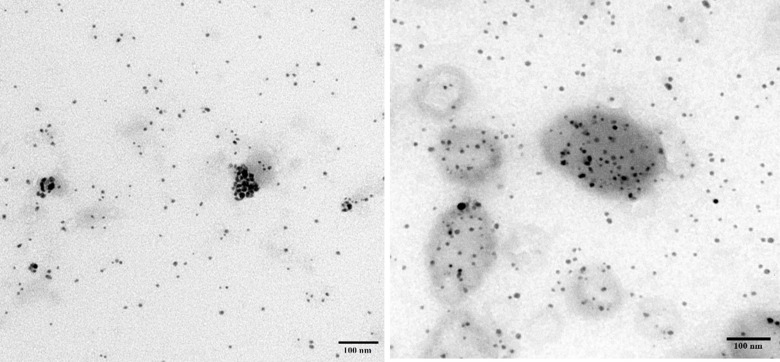
TEM images of AgNPs obtained using aqueous extracts of *A. hierochuntica*.

**FIGURE 3:**
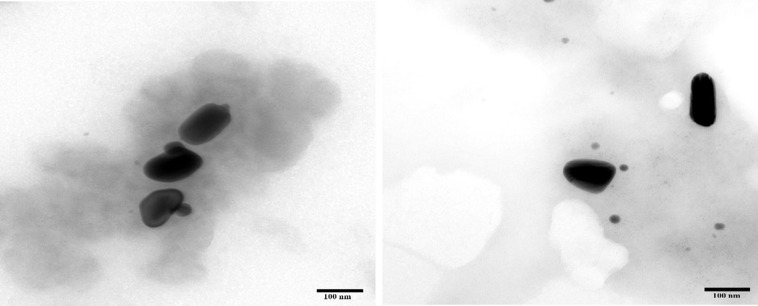
TEM images showing the size of the AgNPs obtained using an aqueous extract of *A. absinthium*.

An-AgNPs and Ar-AgNPs showed mean sizes of 114 ± 2.04 and 125.5 ± 2.5 nm, respectively, as measured via DLS technique (Table 1 and Figures 1A,B). Measurement of the zeta potential returned values of −1.2 and −0.4 mV for An-AgNPs and Ar-AgNPs, respectively. TEM imaging showed that the An-AgNPs were well dispersed and spherical with no significant aggregation (Figure 2). The Ar-AgNPs were semispherical or had no well-defined shape, but no aggregation was observed (Figure 3).

**TABLE 1:** DLS dimensions of NPs size (nm) and potential (mV) and the inhibition zone (mm) of the biogenic NPs as well as the antibiotics tested against different microbes.

Nano material	DLS Mean ± S.D. (nm)	Zeta Potential (m V)	Inhibition Zone Mean ± S.D. (mm)				IC_50_
		
			*E. coli*	*S. aureus*	*P. aeruginosa*	*C. albicans*	MDA-MB-231 cells (μg/mL)
An-AgNPs	114 ± 2.04	−1.2	23.6 ± 3.8	22.4 ± 0.4	23.6 ± 0.2	14.8 ± 0.2	149.2
Ar-AgNPs	125.5 ± 2.5	−0.4	16.8 ± 0.5	16.4 ± 0.7	24.7 ± 0.3	0	585.2
An ions			15.8 ± 1.3	11.7 ± 0.7	24.4 ± 1.2	16.2 ± 2.2	
Bacitracin			0	6 ± 0	0	0	
Tetracycline			0	10 ± 0	15 ± 0	0	
Cefixime			0	10 ± 0	6 ± 0	0	
Ciprofloxacin			0	7 ± 0	35 ± 0	0	
Fluconazole			–	–	–	46.2 ± 0	
Metronidazole			–	–	–	15 ± 0	

*Data represent the mean ± standard deviation of the mean (SD) of three independent experiments.*

**FIGURE 4:**
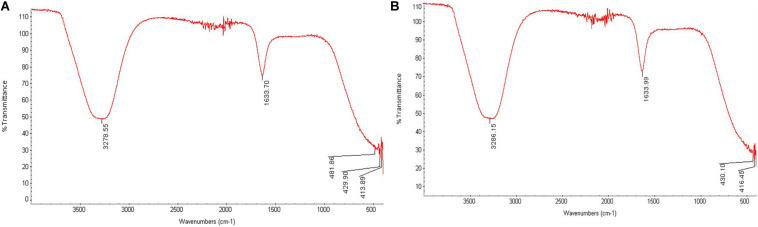
FTIR analysis of An-AgNPs obtained using aqueous extracts of *A. hierochuntica* **(A)** and That for AgNPs obtained using an aqueous extract of *A. absinthium* **(B)**.

Differences in the FTIR data for An-AgNPs and Ar-AgNPs, as shown in Figure 4, provide information about the different biomolecules that might be involved in the reduction of silver ions into AgNPs. Absorption peaks were detected at 3278.55, 1633.70, 481.86, 429.90, and 413.89 cm^–1^ for An-AgNPs (Figure 4A), while absorption peaks were detected at 3286.15, 1633.99, 430.10, and 416.45 cm^–1^ for Ar-AgNPs (Figure 4B).

### Biological Activity and Synergistic Antimicrobial Effects of AgNPs

#### Antimicrobial Activity of An-AgNPs

**FIGURE 5:**
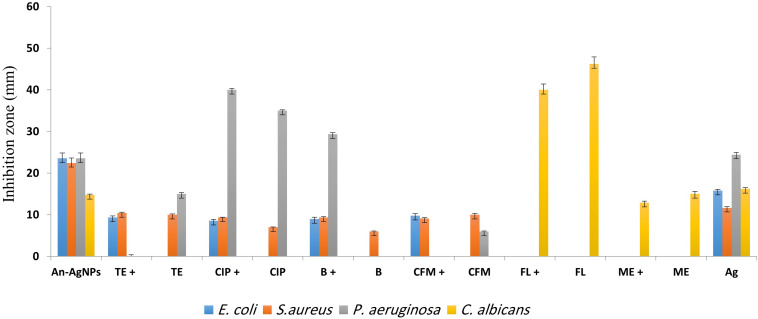
Antibacterial activities of different antibiotics [bacitracin (B), ciprofloxacin (CIP), tetracycline (TE), cefixime (CFM), fluconazole (FL), and metronidazole (ME)] alone and in combination with An-AgNPs at the MIC (50 mg/L). Data are expressed as the means ± SD (*n* = 3 replicates).

Antimicrobial activity was detected at 0.5 μg/well against all tested microorganisms as indicated by the diameters of the inhibition zones (Figure 5). The inhibition zone diameters for *S. aureus, E. coli*, and *P. aeruginosa* were 22.44 ± 0.4, 23.56 ± 3.8, and 23.56 ± 0.2 mm, respectively. As shown in Figure 5, anticandidal activity was observed against *C. Albicans* (14.78 ± 0.2 mm). On the other hand, the activity of An-AgNPs against *P. aeruginosa* and *C. Albicans* did not differ significantly from that of silver ions.

#### The Ability of An-AgNPs to Enhance Antibiotic Efficacy

The activity of An-AgNPs against several microbes was compared to those of standard antibiotics, i.e., bacitracin (BA), ciprofloxacin (CI), tetracycline (TE), and cefixime (CE) as shown in Figure 5. The activity of the An-AgNPs was higher than those of all tested antibiotics against *E. coli* and *S. aureus*. The same observations were made for *P. aeruginosa* with the exception of BA, whose activity was higher than that of the An-AgNPs. For *C. albicans*, An-AgNPs showed lower activity than fluconazole and metronidazole. For *E. coli*, mixtures of An-AgNPs and bacitracin, ciprofloxacin, tetracycline, or cefixime showed substantial synergistic effects relative to the activity of the antibiotic alone. Slight synergistic effects were noted for combinations of An-AgNPs and ciprofloxacin, tetracycline, or cefixime against *S. aureus*. By contrast, a slight decrease in antibiotic activity was observed against *S. aureus* when An-AgNPs were combined with bacitracin.

No antibacterial activity was detected for bacitracin against *P. aeruginosa*; however, a significant synergistic effect was observed for a combination of bacitracin and AgNPs relative to the activity of AgNPs alone. Furthermore, An-AgNPs showed a synergistic effect against *P. aeruginosa* when combined with ciprofloxacin. Combinations of An-AgNPs and tetracycline or cefixime showed no effect on *P. aeruginosa* even though An-AgNPs, tetracycline, and cefixime were active against *P. aeruginosa* when tested separately. These observations suggest antagonistic effects in these antibiotic-AgNP combinations. For *C. albicans*, combinations of An-AgNPs and fluconazole or metronidazole reduced the effects of the anticandidal agents (Figure 5).

#### Antimicrobial Activity of Ar-AgNPs

**FIGURE 6:**
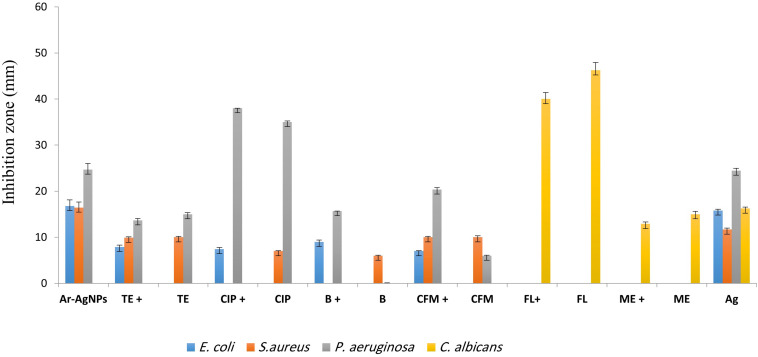
Antibacterial activities of different antibiotics [bacitracin (B), ciprofloxacin (CIP), tetracycline (TE), cefixime (CFM), fluconazole (FL), and metronidazole (ME)] alone and in combination with Ar-AgNPs at the MIC (25–50 mg/L) against the tested microbes. Data are expressed as the means ± SD (*n* = 3 replicates).

Ar-AgNPs at 0.5 μg/well showed potential antibacterial effects against all tested bacteria, as revealed by inhibition zone diameters ranging from 16.44 and 24.67 mm, while no inhibitory activity against *C. Albicans* was observed (Table 1 and Figure 6). Among the pathogenic microorganisms, Ar-AgNPs were more active against *P. aeruginosa* (24.67 mm) followed by *E. coli* (16.78 mm), and *S. aureus* (16.44 mm).

#### The Ability of Ar-AgNPs to Improve Antibiotic Efficacy

For *P. aeruginosa*, Ar-AgNP activity was higher than those of all tested antibiotics except for ciprofloxacin. In this case, the Ar-AgNP activity was approximately 69.4% of that of the antibiotic. Furthermore, combining of Ar-AgNPs with antibiotics resulted in a synergistic effect against *E. coli*; however, this activity was lower than that of Ar-AgNPs alone. For *S. aureus*, no significant differences were observed between the activities of tetracycline or cefixime alone or in combination with Ar-AgNPs; however, addition of Ar-AgNPs to ciprofloxacin or bacitracin suppressed their activities against *S. aureus*, suggesting antagonistic effects. Furthermore, significant synergistic effects on Ar-AgNP activity were observed against *P. aeruginosa* for cefixime, ciprofloxacin, and bacitracin; however, addition of Ar-AgNPs slightly decreased the activity of tetracycline. The activities of combinations of Ar-AgNPs and fluconazole or metronidazole were 86.5 and 85.9%, respectively, of the activities of the anti-fungal agents alone. Ar-AgNPs had no activity against *C. albicans*, but a clear synergistic effect was noted for Ar-AgNPs in combination with fluconazole or metronidazole (Figure 6). The MIC values of the AgNPs ranged from 25 to 50% against all tested microbes (Table 2). The highest activity was recorded for the Ar-AgNPs against *S. aureus* (25% MIC).

#### Morphological Characterization of AgNP-Treated Microbes

**FIGURE 7:**
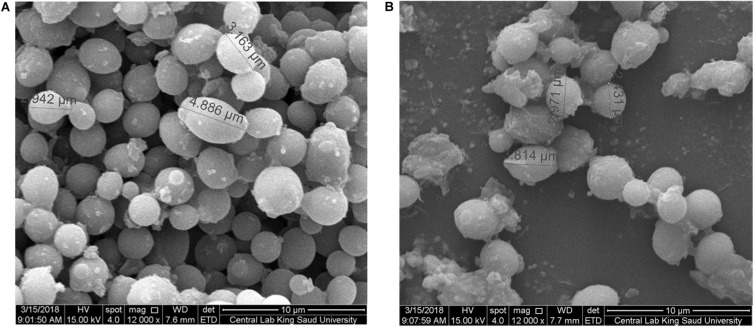
**(A)** SEM image of *C. albicans*. **(B)** SEM image of An-AgNP-treated *C. albicans* showing abnormal cell size and morphology.

**FIGURE 8:**
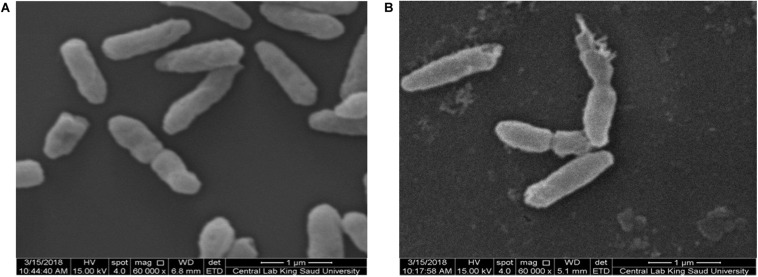
**(A)** SEM image of normal *P. aeruginosa*. **(B)** SEM image of An-AgNP-treated *P. aeruginosa* showing abnormal cell morphology.

An-AgNP-treated *C. albicans* showed abnormal morphology due to their effect by NPs (Figure 7). Variations in morphology, especially cellular elongation, were noticed in An-AgNP-treated *P. aeruginosa* cells compared with untreated control cells (Figure 8).

#### Cytotoxicity

**FIGURE 9:**
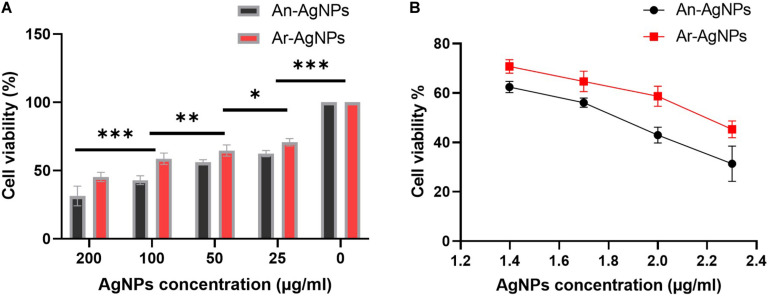
**(A)** IC_50_ values for the AgNPs against MDA-MB231 cell lines after 48 h of treatment. Data are presented as the mean ± SD; *P* values have been calculated against control and **P < 0.001 and *P < 0.01. **(B)** The antiproliferative effect of AgNPs against MDA-MB-231 cells at logarithmic concentration. Data presented as % viability.

To investigate the cytotoxicity of the biogenic AgNPs, cell viability assays were preformed using MDA-MB-231 cells (Table 1). Both An-AgNPs and Ar-AgNPs had dose-dependent inhibitory effects on cell proliferation (Figure 9A). The potency of An-AgNPs was higher (IC_50_ 149.4 μg/mL) compared with that of Ar-AgNPs (IC_50_ 585.2 μg/mL) (Figure 9B).

### Chromatography/Mass Spectrometry (GC-MS) Techniques

The chemical analysis of *A. hierochuntica* and *A. absinthium* were examined by GC-MS analysis and the active constituents are presented in Tables 3, 4. GC-MS analysis of plants under investigation showed similar compounds from both plant origin such as Phenol, 2-methyl-5-(1-methylethyl) and O-xylene. However, from *A. hierochuntica*, 2,2-diethoxypropane, Alkenes and Alkanes such as 1-Pentadecene and Tridecane, respectively, as well as anthraquinone derivatives were also detected. On the other hand, Phenol, 2,4-bis-(1,1-dimethylethyl); TMS (Trimethylsilane); [1,1′-Biphenyl]-4-carbonitrile, 4′-pentyl-; 9-Tetradecen, Amabufotalin, octadecenoic acid methyl ester as well as colchicine were detected from *A. absinthium*.

**TABLE 2:** MIC (%) of aqueous extracts of An-AgNPs and Ar-AgNPs against clinical pathogens.

Treatment	*E. coli*	*S. aureus*	*P. aeruginosa*	*C. albicans*
An-AgNPs	50%	50%	50%	50%
Ar-AgNPs	50%	25%	50%	50%

**TABLE 3:** GC-MS analysis results of *A. hierochuntica* ethanolic extract.

Name of compounds	Formula	Molecular weight	R time (min)
2,2-diethoxypropane	C_7_H_16_O_2_	132	3.268
O-Xylene	C_8_H_10_	106	5.374
2-Cyclohexen-1-one,3,5,5- trimethyl	C_9_H_14_O	138	28.282
p-Pentylacetophenone	C_13_H_18_O	190	28.711
Phenol, 2-methyl-5-(1-methylethyl)-	C_10_H_14_O	150	31.851
1-Pentadecene	C_15_H_3_0	210	32.488
Tridecane	C_13_H_28_	184	39.119
Phenol,2,4-bis(1,1-dimethylethyl)-	C_14_H_22_O	206	45.527
2,5-Cyclohexadiene-1,4-dione,2,6-bis(1,1-dimethylethyl)-	C_14_H_20_O_2_	220	51.680
2-Ethylanthraquinone	C_16_H_12_O_2_	236	69.512

**TABLE 4:** GC-MS analysis results of *A. absinthium* ethanolic extract.

Phenolic Compound	Formula	Molecular weight	R time (min)
O-Xylene	C_8_H_10_	106	5.279
2-Cyclohexen-1-methyl-4-(1-methylethylidene)-	C_10_H_16_	136	13.513
9-Tetradecen-1-ol,(E)-	C_14_H_28_O	212	25.181
TMS (Trimethylsilane)	C_6_H_8_Si	108	28.319
Phenol,2-methyl-5-(1-methylethyl)-	C_10_H_14_O	150	31.889
[1,1′-Biphenyl]-4-carbonitrile, 4′-pentyl-	C_18_H_19_N	249	46.009
2,5-Cyclohexadiene-1,4-dione,2,6-bis(1,1-dimethylethyl)-	C_14_H_20_O_2_	220	51.732
Gamabufotalin	C_24_H_34_O_5_	402	78.808
9-Octadecenoic acid (Z)-, methyl ester	C_19_H_36_O_2_	296	81.881
Colchicine	C_22_H_25_NO_6_	399	89.234

## Discussion

Current developments in the field of nanomedicine have led to great opportunities for nanostructure formation using biogenic agents, providing a simple approach for producing particles with unique characteristics than can combat multidrug-resistant (MDR) bacteria. To the best of our knowledge, that this study is the first to report and analyze AgNP biosynthesis using *A. hierochuntica*, although (del Pilar Rodríguez-Torres et al., 2019) previously tested AgNP production using *A. absinthium*. AgNP biosynthesis proceeded first via a slow color change, after 24 h, the color intensity stabilized, and no further changes were observed, suggesting that color intensity was a time-dependent variable at room temperature. A relationship between the gradual color change and surface plasmon resonance (SPR) can reflect the reduction of silver ions into AgNPs (Algebaly et al., 2020). The exact mechanisms by which AgNPs form with the aid of the biogenic agents is not fully understood; however, plant secondary metabolites may act as reducing and capping agents in this process.

### Characterization of Biogenic AgNPs

Varied sizes for An-AgNPs and Ar-AgNPs suggested that, different extracts can contain different plant metabolites; consistently, variations in their ability to support AgNP formation were indicated by the formation of particles of different sizes when different plant extracts were used for conversion. A recent study reported that AgNPs ranging in size from 2 to 80 nm were produced using *A. absinthium* (del Pilar Rodríguez-Torres et al., 2019) (smaller than those produced via our method described here). This variation might be explained by use of different plant parts because only plant leaves were used in the previous study. Use of different plant parts may lead to variations in the abundance of various biomolecules.

Negative values for zeta potentials for An-AgNPs and Ar-AgNPs, could be related to the organic biomolecules in the plant extract that cap the AgNPs. Negative values might reflect repulsion between the particles, suggesting high degrees of AgNP stability (Bélteky et al., 2019). Use of biological agents for AgNP fabrication normally result in particles with high stability and monodispersity (Iravani et al., 2014).

Regarding TEM imaging for AgNPs, since no washing was performed, the clear and white areas around the AgNPs could be due to biomolecules from the plant extract that cap the synthesized AgNPs. Undefined morphology has also been reported in AgNPs mediated by orange waste (de Barros et al., 2018).

The centers of the nanoparticles were darker than the particle edges, suggesting that organic plant biomolecules may cover and cap the AgNPs, leading to the reduction of silver ions in the AgNPs (Khandel et al., 2018). In general, the TEM data revealed smaller particles compared with the size predicted by DSL data, likely because impurities (e.g., bio-active molecules derived from the biogenic agents) surround the AgNPs. This property would have a stronger effect on the DLS readings due to the higher quantity of AgNP solution required for the measurement in contrast to the quantity normally used for TEM. Some cellular biomolecules have been shown to affect AgNP fabrication, including carbohydrates, amino acids, enzymes, proteins, and pigments (El-Naggar et al., 2016). Concentration of such biomolecules might be the main reason on the divergence of detected peaks using DLS. Furthermore, variations between NPs size detected by DLS and TEM technique is expected since the principles are varied for each instrument (Khandel et al., 2018). In general, the shape and size variation observed here might be expected since various reduction routes for silver ions are likely to exist in plant extracts due to the presence of multiple reducing and capping agents, which was verified by the FTIR analysis described below. FTIR is used to detect organic and inorganic compounds based on their infrared spectra (Leela and Anchana, 2017).

The spectra for both An-AgNPs and Ar-AgNPs ranged from 3300 to 3,500 cm^–1^, suggesting the presence of polyphenolic hydroxyl (–OH) groups and N–H bond stretching in amine groups (Siddiqi et al., 2018). Absorption between 1,600 and 1,650 cm^–1^, which can be attributed to the amide I band associated with carbonyl C = O stretching in proteins, was detected for both types of AgNPs (Khandel et al., 2018). Peaks in the 400 to 450 cm^–1^ range can be related to silver ions in both AgNPs. Polyphenolic and amide I absorption bands were noted for both types of AgNPs; however, such compounds might vary in concentration or type as indicated by slight differences in the FTIR spectra. The presence of peaks corresponding to hydroxyl and amide groups suggests that carbohydrates and proteins are involved in the reducing and capping of the AgNPs prepared using the two plant extracts. These findings suggest that these AgNPs are both safe and stable.

### Biological Activity and Synergistic Antimicrobial Effects of AgNPs

#### Antimicrobial Activity of An-AgNPs

To determine whether the conversion of silver ions into AgNPs using plant extracts affected the biological characteristics of the biogenic-AgNPs, their effects on various microbes were tested. The activity of the An-AgNPs was higher than that of silver ions against *E. coli* and *S. aureus*, suggesting that their small particle size facilitated entry into the cells where they were more likely to induce damage (Pal et al., 2007). Use of AgNPs as antiseptics might be fueled by their broad-spectrum activity (Jones et al., 2004); however, interactions between sulfur groups in cell wall proteins and silver ions might specifically disrupt the bacterial cell wall (Rai et al., 2012; Abbaszadegan et al., 2015). The observed antibacterial effects of positively charged An-AgNPs might also be related to cell wall disruption driven by their ability to electrostatically adhere to the negatively charged bacterial membranes (Rai et al., 2012; Abbaszadegan et al., 2015). The catalytic oxidation of silver metal and monovalent silver ion interactions underly the bactericidal action of AgNPs (Abo-Shama et al., 2020).

#### The Ability of An-AgNPs to Enhance Antibiotic Efficacy

AgNPs are less likely than antibiotics to promote microbial resistance acquisition (Jones et al., 2004); therefore, the activity of An-AgNPs against several microbes was compared to those of standard antibiotics. Due to the increasing prevalence of antibiotic-resistant microorganisms, which threaten human health globally, we explored the efficacy of combinations of antibiotics and biogenic AgNPs. Microbial antibiotic resistance is rising more rapidly than silver resistance because acquisition of metal resistance requires accumulation of multiple mutations due to the multiple targets of metals (Herrera et al., 2001). In light of this trend, we examined potential synergism between the effects of AgNPs and antibiotics and the anticandidal agents fluconazole and metronidazole. Each antibiotic was mixed with AgNPs, and the antimicrobial effects of each mixture were tested.

A recent study confirmed the synergistic effects of combinations of some antibiotics and AgNPs against *E. coli* and *Salmonella* spp. (Abo-Shama et al., 2020). *Salmonella typhimurium* was synergistically inhibited by combinations of tetracycline and AgNPs and neomycin and AgNPs (McShan et al., 2015).

#### Antimicrobial Activity of Ar-AgNPs

Ar-AgNPs were more active than silver ions against the tested bacterial species, no activity was observed against *C. albicans*. By contrast, AgNPs prepared using *A. absinthium* have been shown to have significant activity against pathogenic yeasts of the *Candida* genus (del Pilar Rodríguez-Torres et al., 2019). This discrepancy might indicate that the smaller AgNPs prepared using *A. absinthium* in the earlier study (relative to the AgNPs prepared in our study) might confer increased activity against *Candida* spp. or that the activity of AgNPs fabricated using *A. absinthium* might be species specific. AgNPs and ZnNPs prepared using *Ulva fasciata* alga showed no anticandidal activity (Abo-Shama et al., 2020).

#### The Ability of Ar-AgNPs to Improve Antibiotic Efficacy

Combinations of antibiotics and AgNPs have been shown to exert an antibiofilm effect against mature bacterial biofilms (Hwang et al., 2012a). Furthermore, addition of AgNPs to antibiotics promotes an increase in the local antibiotic concentration around the site of action in bacteria, thus facilitating a strong antibiotic-microbe interaction (Allahverdiyev et al., 2011). Sharma et al. (2016) demonstrated synergistic effects of ZnO nanoparticles combined with ciprofloxacin against *S. aureus*, *E. coli*, and *Klebsiella pneumoniae*. Increased penetration of the antibiotic active ingredients facilitated by nanoparticles could explain the synergistic effect of AgNP-antibiotic combinations; however, antagonistic effects were also observed. Such effects could be related, at least to some extent, to effects of the biomolecules capping the AgNPs; for example, these molecules might interfere with antibiotic components and limit their availability.

In general, the activity of the An-AgNPs was higher than that of the Ar-AgNPs against *E. coli*, *S. aureus*, and *C. albicans*, while no significant differences were observed between their activities against *P. aeruginosa*. These observations might be related to the smaller particle size of the An-AgNPs compared with that of Ar-AgNPs, which would result in a higher surface-to-volume ratio. This effect might support nanoparticle entry into the cells via the larger contact area with the microbial cell wall. The tolerance level for all tested AgNPs was bactericidal since the MBC:MIC ratio was ≤2 (Long, 2017).

#### Morphological Characterization of AgNP-Treated Microbes

To elucidate the antimicrobial mechanism of An-AgNPs, *P. aeruginosa* and *C. albicans* were treated with biogenic An-AgNPs and then observed under SEM to identify any differences in cell shape. TEM imaging has previously confirmed penetration and intracellular accumulation of AgNPs prepared using *Syzygium cumini* seed extract in *C. albicans* in addition to cell wall damage (Jalal et al., 2019), such information could support our findings in the abnormal morphology and cell elongation of An-AgNP-treated *C. albicans*, and *P. aeruginosa* cells, respectively. The precise antibacterial and anticandidal mechanisms of AgNPs are not fully understood. Various modes of action have been proposed, e.g., (1) induction of DNA and cytoplasmic leakage (Jun et al., 2000), (2) creation of holes in the cell membrane to induce death in *C. albicans* (Kim et al., 2009), and (3) induction of ROS production (Hwang et al., 2012b). Similar trends have been reported elsewhere (Gopinath et al., 2015; Hamouda et al., 2019).

The present observations of potential inhibitory activities against several pathogenic microbes suggests that An-AgNPs and Ar-AgNPs could be used as alternatives to antibiotics in various medical applications.

### Cytotoxicity

To investigate the cytotoxicity of the biogenic AgNPs, cell viability assays were preformed using MDA-MB-231 cells. In general, anticancer activity in AgNPs prepared using green synthesis has been well documented (Jeyaraj et al., 2013; Paul et al., 2015; Al-Sheddi et al., 2018; Algebaly et al., 2020); however, different IC_50_ values and variations in cytotoxicity (which might be related to cell line and AgNP size, shape, and concentration) have been reported. The higher efficacy of An-AgNPs compared to Ar-AgNPs against MDA-MB-231 cells might be due to their smaller particle size; however, AgNPs generally have a high affinity for cell membranes that facilitates cell entry. Organic biomolecules derived from the plant extract that cap the AgNPs might also enhance their stability and facilitate cellular entry by stimulating cellular uptake. In general, various modes of action might enhance cancer cell suppression, including ROS generation, which leads to DNA damage and apoptosis via consequential caspase-3 activation (Siddiqi et al., 2018).

### Chromatography/Mass Spectrometry (GC-MS) Techniques

Furthermore, the GC-MS investigation in the current study approved the incidence of various phytochemicals that could be responsible for the biological activity of AgNPs. Such molecules involving Phenolic compounds, the well-known antioxidant molecules with different biological activities. Antioxidant and antimicrobial effects were noted for the aromatic hydrocarbons, O-xylene (Tiwari et al., 2016) that detected for both currently investigated plants. A 2,2-diethoxypropane could has a main role as antimicrobial agent since it was also detected in *Mentha cervina* and *Origanum vulgare* essential oils that showed antimicrobial ability (Helal et al., 2019). Furthermore, anthraquinones could also contribute to the biological activity of *A. hierochuntica*. Antibacterial, anti-inflammatory and antioxidant effects were noted for *Ceratotheca triloba* from which anthraquinones were detected (Mohanlall and Odhav, 2013). Amabufotalin is a well-known cytotoxic agent (Yu et al., 2014). Presence of octadecenoic acid methyl ester and colchicine might also contribute to the activity of *A. absinthium*. Antimicrobial activity was noted for J. curcas leaf extracts that contain a component octadecenoic acid methyl ester (Asghar and Choudahry, 2011). The main alkaloid, colchicine, had cytotoxic activity (Kurek, 2018). Such Phenolic and other phytochemicals could be also essential in nanoparticle formation (Ouerghemmi et al., 2017).

## Conclusion

The use of biogenic agents for nanoparticle formation is a remarkable advance in nanoscience that yields safe and stable nanostructures capped with biomolecules. The biogenic AgNPs in the current investigation were prepared using aqueous extracts from *A. hierochuntica* L. (Kaff Maryam) and *A. absinthium*. This method yielded biogenic nanoparticles with diameters ranging from 114 to 125.5 nm based on our DLS results. The current approach is simple, economical, and effective for producing AgNPs at room temperature using safe materials without the production of toxic byproducts. The AgNPs produced using both plant extracts demonstrated antimicrobial effects against multiple microorganisms, strong synergistic antibacterial activity in combination with some antibiotics, as well as cytotoxic effects against MDA-MB-231 cells. Based on our findings, we propose that the use of plant extracts for nanoparticle fabrication might be useful for large-scale production of nanomaterials. Combinations of AgNPs and antibiotics had synergistic antimicrobial effects, which might help to explain their mechanism of action. Furthermore, AgNPs capped and reduced by biomolecules such as carbohydrates and proteins are safe and stable nanoparticles that might be useful in antimicrobial drug formulations, making them potentially valuable for the pharmaceutical industry. Various levels of toxicity might be expected in different AgNP applications; therefore, it is important to carefully control and optimize the conditions needed to maintain an appropriate and safe concentration.

## Data Availability Statement

The raw data supporting the conclusions of this article will be made available by the authors, without undue reservation.

## Author Contributions

KA: conceptualization, funding acquisition, formal analysis, methodology, supervision, writing – original draft, writing, review, and editing. AM: conceptualization, formal analysis, methodology, visualization, writing – original draft, writing, review, and editing. Both authors contributed to the article and approved the submitted version.

## Conflict of Interest

The authors declare that the research was conducted in the absence of any commercial or financial relationships that could be constructed as a potential conflict of interest.

## Supplementary Material

Supplementary File 1 **(A)** *A. hierochuntica* L. (Kaff Maryam) extract. **(B)** Silver nanoparticles prepared using *A. hierochuntica* L. (Kaff Maryam) extract. **(C)** *A. absinthium* extract. **(D)** Silver nanoparticles prepared using *A. absinthium* extract.
