# Supplementary figures and images for: Synergistic and Antagonistic Effects of Biogenic Silver Nanoparticles in Combination With Antibiotics Against Some Pathogenic Microbes
Source: Front Bioeng Biotechnol. 2021 Apr 20;9:652362. doi: 10.3389/fbioe.2021.652362 (PMC8093520; doi:10.3389/fbioe.2021.652362)

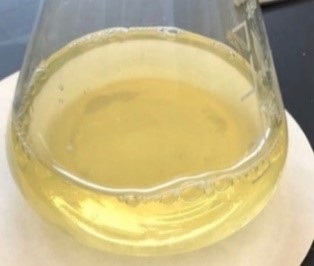

Supplement: Supplementary File 1 — (A) A. hierochuntica L. (Kaff Maryam) extract. (B) Silver nanoparticles prepared using A. hierochuntica L. (Kaff Maryam) extract. (C) A. absinthium extract. (D) Silver nanoparticles prepared using A. absinthium extract. [file Image_1.jpg]

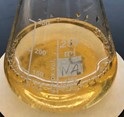

Supplement: Supplementary file 2 [file Image_2.jpg]

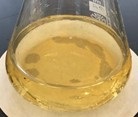

Supplement: Supplementary file 3 [file Image_3.jpg]

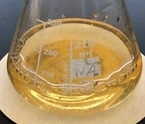

Supplement: Supplementary file 4 [file Image_4.jpg]
